# Correlation of greyzone fibrosis compared to troponin T and late gadolinium enhancement with survival and ejection fraction in patients after acute myocardial infarction

**DOI:** 10.1007/s00392-024-02536-w

**Published:** 2024-09-04

**Authors:** Ramona Schmitt, Clara Staats, Klaus Kaier, Christoph Ahlgrim, Manuel Hein, Johannes Brado, Philipp Steinhoff, Hannah Billig, Martin Soschynski, Tobias Krauss, Christopher L. Schlett, Dirk Westermann, Franz-Josef Neumann, Philipp Ruile, Philipp Breitbart

**Affiliations:** 1 Department of Cardiology and Angiology, Medical Center-University of Freiburg, Faculty of Medicine, University of Freiburg, Südring 15, 79189 Bad Krozingen, Germany; 2 Institute of Medical Biometry and Statistics, Faculty of Medicine and Medical Center, University of Freiburg, Freiburg, Germany; 3 Department of Cardiology, University Hospital Bonn, Venusberg-Campus 1, 53127 Bonn, Germany; 4 Department of Diagnostic and Interventional Radiology, Medical Center-University of Freiburg, Faculty of Medicine, University of Freiburg, 79106 Freiburg, Germany

**Keywords:** Acute myocardial infarction, Greyzone, Cardiovascular magnetic resonance imaging

## Abstract

**Aims:**

To quantify greyzone fibrosis (GZF) in patients after acute myocardial infarction (MI) and to evaluate its correlation with MI-free survival and improvements in left ventricular ejection fraction (LVEF) compared with the established risk factors high-sensitivity cardiac troponin T (hs-cTnT) and Late Gadolinium Enhancement (LGE).

**Methods and results:**

The study involved 176 patients who experienced acute MI and underwent cardiac magnetic resonance (CMR) prior to hospital discharge, followed by a second CMR on average six months later. LGE was quantified in both examinations, a separate analysis of the GZF was conducted only in the follow-up CMR after resolution of the initial infarct edema. LVEF was measured in both CMR. hs-cTnT levels were assessed at hospital admission, as well as 8, 16, 24, 48 and 72 h after coronary intervention. Telephone follow-ups were conducted annually for up to 8 years. LGE measurements showed better correlation with MI-free survival (Harrell’s C of 0.711 of LGE mass) compared to GZF (0.579 of GZF mass). Additionally, hs-cTnT outperformed GZF (Harrell’s *C* of 0.645). As an univariable predictor for MI-free survival, only hs-cTnT reached significance (*p* < 0.05). With regard to improvements in ejection fraction, both hs-cTnT and LGE measurements showed acceptable correlation with improvement in ejection fraction (*p* < 0.05), while GZF measurements showed no correlation (*p* > 0.5).

**Conclusions:**

In CMR, the assessment of GZF demonstrated inferior p correlation compared to hs-cTnT and LGE in patients after acute MI with respect to the endpoint of MI-free survival. Furthermore, GZF showed no correlation with the improvement of LVEF.

**Graphical abstract:**

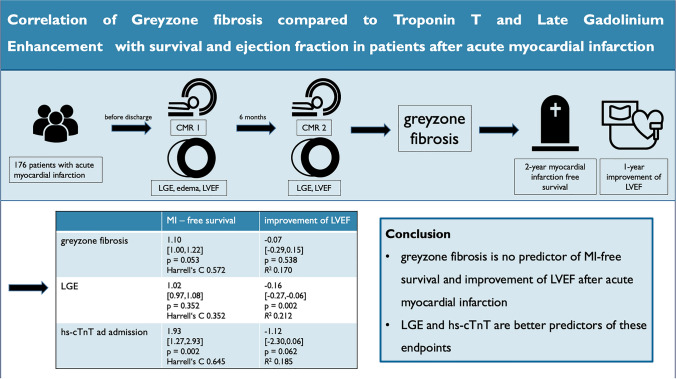

**Supplementary Information:**

The online version contains supplementary material available at 10.1007/s00392-024-02536-w.

## Introduction

Currently, risk stratification for patients with myocardial infarction (MI) or ischemic heart disease is mostly based on left ventricular ejection fraction (LVEF), e.g., the recommendation for an implantable cardioverter-defibrillator (ICD) [1, 2].

However, other risk factors like high levels of high-sensitivity cardiac troponin T (hs-cTnT), presence of myocardial fibrosis (Late Gadolinium Enhancement, LGE), hypertension or diabetes for increased mortality after MI have also been described [3–5]. So-called greyzone fibrosis (GZF) can be detected by cardiac magnetic resonance (CMR) and consists of myocardial fibrosis and viable myocardium [6]. It is described as a risk factor for life-threatening arrhythmias, such as ventricular fibrillation in patients with coronary artery disease [3, 6].

So far, data about the correlation between the amount of GZF occurring after MI and long-term clinical parameters are scarce. Thus, the purpose of this study was to quantify GZF and to evaluate its correlation with survival and improvements in LVEF in a well-characterized cohort with an index event. The study furthermore aimed at comparing these correlations with those of the established risk factors hs-cTnT and Late Gadolinium Enhancement.

## Methods

This retrospective study included patients who experienced myocardial infarction type I (ST-segment elevation and non-ST-segment elevation myocardial infarction [7]) from September 2014 to November 2019. These patients underwent CMR prior to hospital discharge, followed by a second CMR on average six months later. Patients with poor image quality at CMR were excluded from the analyses. All clinical data and follow-up (FU) information were sourced from our institutional database.

High-sensitivity cardiac troponin T (hs-cTnT) was measured (cobas pro, Roche Germany Holding GmbH, cutoff value < 0.014 ng/ml) at admission, 8, 16, 24, 48 and 72 h after PCI and patients were monitored using a standardized follow-up protocol, including annual phone calls for up to 8 years to determine adverse events such as death, myocardial infarction, stroke, bleeding, and hospitalizations. All patients gave their written informed consent for the anonymized use of clinical, procedural and follow-up data at the time of the intervention. This study was approved by the institutional review board and complied with the Declaration of Helsinki.

### Cardiac magnetic resonance imaging

All CMR-examinations were performed on a 3.0 Tesla scanner (Siemens Magnetom Skyra, Siemens Healthineers, Forchheim, Germany) with patients placed in supine position and using a cardiac coil. Images were acquired at end-expiratory breath hold. A bolus of contrast agent was applied (0.2 ml/kg bodyweight, Magnevist^®^, Bayer Pharma, Berlin, Germany). 5 min after the bolus, retrospectively gated contrast-enhanced steady-state free precision (SSFP) cine images in short-axis (SAX) stack covering the left ventricle from the base to apex, 2-, 3- and 4-chamber view were acquired. Image parameters for SSFP cines were: TE 1.4 ms; TR 2.9 ms; flip angle 60°; image resolution 1.5 × 1.5 × 8 mm; slice gap 0 mm. No parallel imaging was performed to maximize the signal-to-noise ratio (SNR). 15 min after contrast injection, late gadolinium enhancement (LGE) images were acquired in the same planes as cine images with a phase-sensitive inversion-recovery sequence (TE 3.3 ms, TR 7.0 ms, TI 250–500 ms to null the myocardium, 8 mm slice, no gap, matrix 256 × 192).

### Image analysis

Image analysis was performed with dedicated post-processing workstations (syngo.via, Siemens Healthineers AG, Forchheim, Germany; CVI42, Circle Cardiovascular Imaging Inc, Calgary, AB, Canada) by two experienced readers (P.B. and P.R. with > 3 years of experience in CMR and both certified with the highest degree in CMR of the German Cardiac Society) independently.

Volume measurements, LVEF, stroke volume, cardiac index and myocardial mass were semi-automatically assessed using the SSFP-cine images. For all analyses, the endocardial and epicardial borders of the left ventricle were manually traced in all short-axis slices in end-diastole and end-systole. The papillary muscles were excluded from the myocardium. Improvement of LVEF was defined as the delta between LVEF in CMR 1 and in CMR 2. As a parameter for left ventricular remodeling we defined the delta between left ventricular end-diastolic volume (LVEDV) in CMR 1 and in CMR 2.

**Fig. 1:**
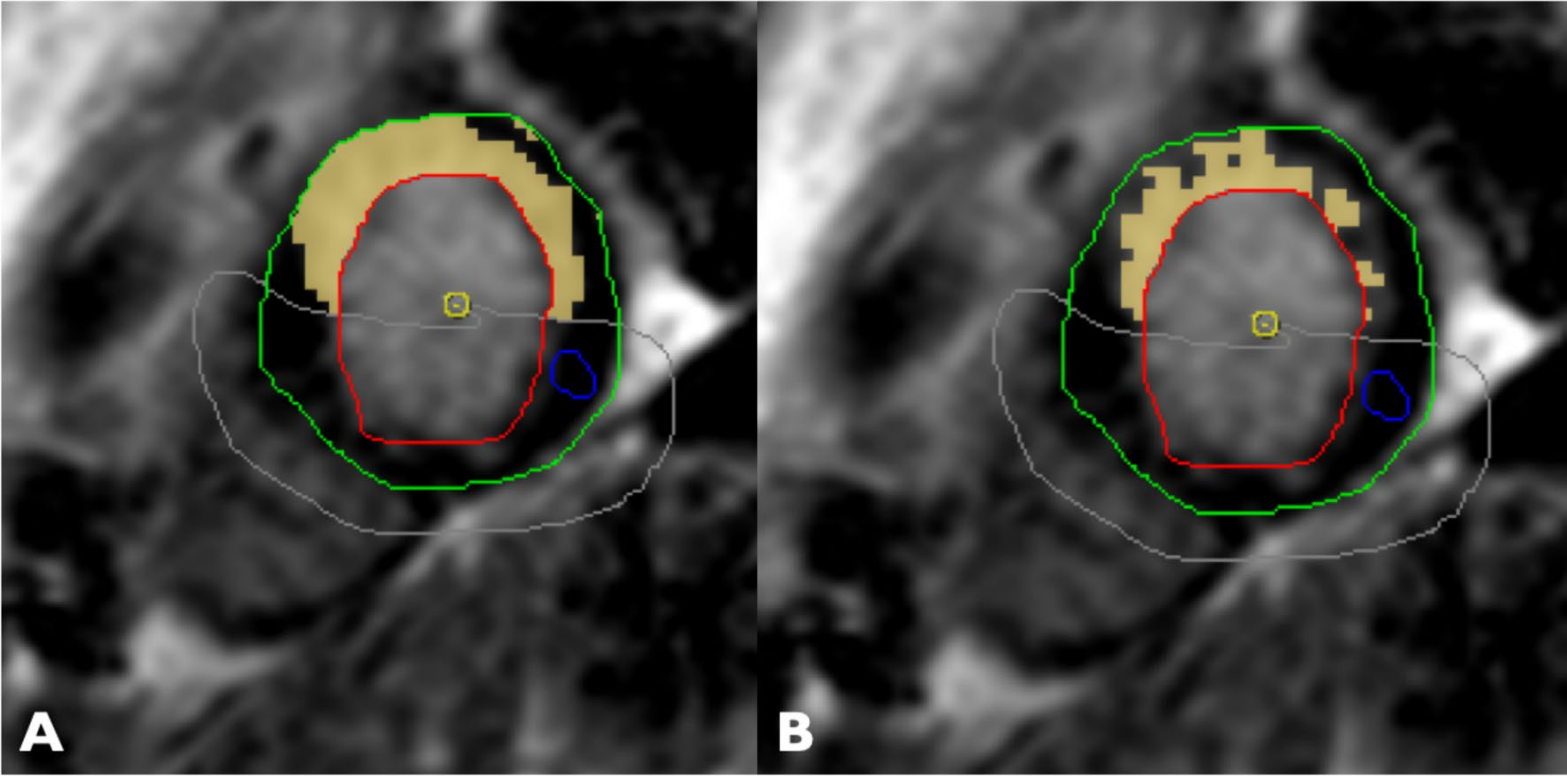
Image analysis—quantification of the infarct area: **A** SI > 3 SD and **B** SI > 5 SD. Yellow—infarct area, green—epicardial border, red—endocardial border, blue—remote myocardium, grey—visual adjustment/exclusion line. *SI* signal intensity, *SD* standard deviation

In order to quantify the myocardial edema in the contrast-enhanced SSFP-cine images [8] of first CMR, a semiautomatic delineation using signal-intensity (SI) thresholds of the hyperintense, edematous region (SI > 2 SD exceeding the mean SI of remote myocardium) was performed in all short-axis slices (in systole). The infarct area was semi-automatically assessed in the short-axis LGE images in both CMR (SI > 5 SD exceeding the mean SI of remote myocardium) (Fig. 1). A hypointense signal within the area of LGE representing microvascular obstruction, if present, was included in the analysis. All automatically assessed areas were visually controlled and adjusted if necessary.

For GZF analysis, the mean SI of remote myocardium was adopted to 3 SD SI and the area of enhanced myocardium was semi-automatically assessed. GZF was calculated as follows: 3 SD SI—LGE.

### Statistical analysis

All statistical analyses were performed using Stata (StataCorp LCC, Texas, USA, version 18).

Categorical variables are expressed as frequencies and percentages, continuous variables as mean and standard deviation (SD) or median with interquartile range (IQR). For MI-free survival, univariable and bivariable Cox regression models were conducted, and Harrell’s *C* and Royston and Sauerbrei’s *D* were calculated as discrimination measures and compared across different potential predictors. For improvements in ejection fraction, univariable and bivariable linear regression models were conducted and the coefficients of determination *R*^2^ were calculated and compared. A value of *p* < 0.05 was considered as statistically significant. Observer agreement was assessed using the intraclass correlation coefficient, with values above 0.90 indicating excellent reliability.

## Results

176 patients (18.2% female, 63.3 ± 11.4 years) with acute myocardial infarction (114 with ST-segment elevation myocardial infarction (STEMI), 62 with non-ST-segment elevation myocardial infarction (NSTEMI)) were included in this study. Mean time between MI and CMR 1 was 3 ± 1 days and between MI and CMR 2 191 ± 65 days. Median time between CMR 1 and CMR 2 was 186 days [interquartile range, IQR, 181; 194].

Median hs-cTnT-level at admission was 0.240 ng/ml [0.072; 0.665] and median peak hs-cTnT-level was 2.835 ng/ml [1.043; 5.212]. CMR analyses showed a mean LVEF of 49.8 ± 9.7% at admission (= CMR 1) and of 55.1 ± 9.7% at follow-up (= CMR 2). Interobserver reliability was high for CMR-measurement of LVEF [intraclass correlation coefficient 0.973 (95% confidence interval [CI]: 0.912–0.991)].

Mean LGE mass was 14.21 ± 11.98 g, mean GZF 4.77 ± 2.89 g. Patients with STEMI had significant higher levels of hs-cTnT at any timepoint as well as higher amounts of LGE (*p* < 0.001 each) than patients with NSTEMI. There were no significant differences between patients with STEMI and NSTEMI regarding the amount of myocardial edema (*p* = 0.125) and GZF (*p* = 0.253).

All baseline characteristics including the treatment of the acute myocardial infarction are summarized in Table 1, and hs-cTnT-levels and CMR analyses are summarized in Table 2.

**Table 1:** Baseline characteristics and treatment results of the study population

	Summary
*N*	176
Age (years)	63.3 (11.4)
Sex	
Male	144 (81.8%)
Female	32 (18.2%)
Cardiovascular risk factors	
Hypertension	103 (58.5%)
Smoking history	55 (31.3%)
Hypercholesterinemia	96 (54.5%)
Family history of MI	45 (25.6%)
Diabetes	30 (17.0%)
Previous myocardial infarction	8 (4.5%)
Previous PCI	19 (10.8%)
Previous coronary bypass surgery	3 (1.7%)
Type of myocardial infarction	
ST-elevation	114 (64.8%)
Non-ST-elevation	62 (35.2%)
Treatment of myocardial infarction	
Stent implantation	172 (97.7%)
Drug-eluting balloon	1 (0.6%)
Non-drug eluting balloon	3 (1.7%)
Remaining stenosis	
Yes	36 (20.5%)
Same coronary segment	1 (2.8%)
Same coronary artery	7 (19.4%)
Other coronary artery	28 (77.8%)
No	140 (79.5%)
TIMI flow	
TIMI 0	1 (0.6%)
TIMI 1	3 (1.7%)
TIMI 2	53 (30.1%)
TIMI 3	119 (67.6%)
Myocardial blush grade	
Grade 0	2 (1.1%)
Grade 1	16 (9.1%)
Grade 2	41 (23.3%)
Grade 3	117 (66.5%)
Medical treatment at discharge	
Aspirin	158 (89.8%)
P2Y_12_ receptor inhibitors	176 (100.0%)
Oral anticoagulation	29 (16.5%)
ACE inhibitor	136 (77.3%)
AT1 receptor antagonist	28 (15.9%)
Betablocker	150 (85.2%)
Mineralocorticoid receptor antagonist	38 (21.6%)
ARNI	0 (0.0%)
SGLT2 inhibitor	2 (1.1%)
Non-sustained ventricular tachycardia during hospitalization	
Yes	24 (13.6%)
No	152 (86.4%)

Values are mean ± standard deviation or frequencies and percentages

*ACE* angiotensin-converting enzyme, *ARNI* angiotensin-receptor neprilysin inhibitor, *AT1* angiotensin 1, *MI* myocardial infarction, *PCI* percutaneous coronary intervention, *SGLT2* sodium-glucose cotransport-2, *TIMI* thrombolysis in myocardial infarction

**Table 2:** hs-cTnT-levels and CMR results of the study population

	Summary
hs-cTnT at admission (ng/ml)	0.240 [0.072; 0.665]
hs-cTnT 8 h after PCI (ng/ml)	2.480 [0.841; 4.833]
hs-cTnT 16 h after PCI (ng/ml)	1.990 [0.897; 3.608]
hs-cTnT 24 h after PCI (ng/ml)	1.780 [0.740; 3.375]
hs-cTnT 48 h after PCI (ng/ml)	1.800 [0.869;3.405]
hs-cTnT 72 h after PCI (ng/ml)	] 2.220 [0.923; 3.470]
Peak hs-cTnT (ng/ml)	2.835 [1.043; 5.213]
Time between MI and CMR (days)	
CMR 1	3 (1.0)
CMR 2	191 (65.0)
Time between CMR 1 and CMR 2 (days)	186 [181,194]
LGE mass (g)	14.21 ± 11.98
Myocardial edema (g)	35.00 ± 29.75
GZF mass (g)	4.77 ± 2.89
LVEF CMR 1 (%)	49.8 ± 9.7
LVEF CMR 2 (%)	55.1 ± 9.7
LVEDV CMR 1 (ml)	175.3 ± 44.1
LVEDV CMR 2 (ml)	176.9 ± 44.3
MVO CMR 1	
Yes	38 (21.6%)
No	155 (88.1%)

Values are mean ± standard deviation, median with interquartile range or frequencies and percentages

*CMR* cardiac magnetic resonance, *GZF* greyzone fibrosis, *LVEDV* left ventricular end-diastolic volume, *LVEF* left ventricular ejection fraction, *hs-cTnT* high-sensitive cardiac troponin T, *LGE* late gadolinium enhancement, *MVO* microvascular obstruction

### Follow-up

**Fig. 2:**
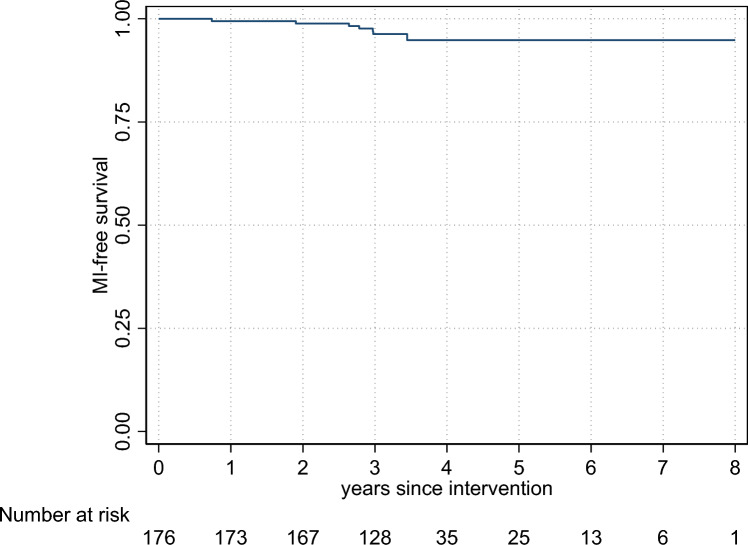
Myocardial-infarction-free survival in the follow-up period. Graph shows the myocardial infarction (MI)-free survival of the study cohort in the follow-up period after the intervention

No patient died or suffered a second myocardial infarction in the period between CMR 1 and CMR 2. During the median follow-up period of 1132 days [1093; 1331], six patients died (3.4%, 3 from neurological diseases, 1 from pneumonia, 1 from heart failure and 1 from myocardial infarction). Seven patients suffered a second myocardial infarction (4.0%, Fig. 2). During follow-up, 158 patients (89.8%) were hospitalized for a planned coronary angiography. All follow-up data are summarized in Table 3.

**Table 3:** Follow-up of the study population

	Summary
*N*	176
Follow-up period (days)	1132 [1093; 1331]
Re-hospitalization	174 (98.9%)
Planned coronary angiography	158 (89.8%)
Acute coronary syndrome	5 (2.8%)
Other cardiac hospitalization	3 (1.7%)
Extracardiac hospitalization	1 (0.6%)
Coronary intervention during follow-up	
None	109 (61.9%)
Same coronary segment	10 (5.7%)
Same coronary artery	13 (7.4%)
Other coronary artery	42 (23.9%)
Death within follow-up	
Yes	6 (3.4%)
No	170 (96.6%)
MI-free survival	
Yes	169 (96.0%)
No	7 (4.0%)
Stroke within follow-up	
Yes	3 (1.7%)
No	173 (98.3%)
Bleeding within follow-up	
Yes	3 (1.7%)
No	173 (98.3%)

Values are median with interquartile range or frequencies and percentages

*MI* myocardial infarction

Regression analyses and analyses for predictive power showed that LGE measurements were associated with better correlation (Harrell’s *C* of 0.711 of LGE mass) compared to GZF (0.579 GZF) regarding MI-free survival. hs-cTnT at admission also performed better (Harrell’s *C* of 0.645) than GZF and peak hs-cTnT or hs-cTnT 8, 16, 24, 48 and 72 h after PCI. As an univariable predictor for MI-free survival, only hs-cTnT at admission reached significance (*p* = 0.002) (Table 4, supplementary tables 1, 2 and 3).

**Table 4:** Results of univariable Cox model on the endpoint MI-free survival

	1	2	3	4	5	6	7	8	9	10
	1.93									
hs-cTnT at admission	[1.27, 2.93]									
	(0.002)									
		1.04								
hs-cTnT 8 h after PCI		[0.85, 1.26]								
		(0.706)								
			1.11							
hs-cTnT 16 h after PCI			[0.87, 1.41]							
			(0.410)							
				1.21						
hs-cTnT 24 h after PCI				[0.94, 1.56]						
				(0.141)						
					1.00					
hs-cTnT 48 h after PCI					[1.00, 1.00]					
					(0.944)					
						1.01				
hs-cTnT 72 h after PCI						[0.66, 1.55]				
						(0.960)				
							1.00			
Peak hs-cTnT							[1.00, 1.00]			
							0.953			
								1.02		
LGE mass								[0.97, 1.08]		
								(0.359)		
									1.18	
GZF mass									[1.01, 1.39]	
									(0.037)	
										1.60
MVO mass										[0.29, 8.72]
										(0.589)
Observations	176	172	166	163	147	94	176	176	176	160
Harrell’s C (~ AUC)	0.645	0.494	0.532	0.569	0.355	0.566	0.495	0.711	0.587	0.562
Royston and Sauerbrei’s *D* (~ *R*^2^)	0.260	− 0.004	0.000	0.040	− 0.113	− 0.028	0.002	0.139	0.152	0.044

Hazard ratios; 95% confidence intervals in brackets; *p* values in parentheses

*GZF* greyzone fibrosis, *hs-cTnT* high-sensitive cardiac Troponin T, *LGE* late gadolinium enhancement, *MI* myocardial infarction, *MVO* microvascular obstruction, *PCI* percutaneous coronary intervention

With regard to improvements in ejection fraction, hs-cTnT, LGE and MVO measurements as well as the presence of STEMI showed acceptable correlation with improvement in ejection fraction (*p* < 0.05), but GZF measurements showed no correlation (*p* > 0.05) (Table 5). Regarding left ventricular remodeling, hs-cTnT, GZF, LGE and MVO showed acceptable correlation with improvement in LVEDV (*p* < 0.05), but GZF showed worse *R* squared values than LGE and hs-cTnT (GZF < 0.2, hs-cTnT and LGE > 0.2) (supplementary Table 4).

**Table 5:** Results of univariable [model 1] and bivariable [model 2 to 12] linear regression models on the endpoint delta LVEF (= LVEF CMR 2 minus EF CMR 1)

	1	2	3	4	5	6	7	8	9	10	11	12
	− 0.33	− 0.35	− 0.44	− 0.36	− 0.43	− 0.35	− 0.36	− 0.34	− 0.44	− 0.35	− 0.38	− 0.35
Delta LVEF	[− 0.45, − 0.22]	[− 0.47, − 0.24]	[− 0.56, − 0.33]	[− 0.47, − 0.25]	[− 0.54, − 0.31]	[− 0.48, − 0.23]	[− 0.53, − 0.19]	[− 0.45, − 0.23]	[− 0.57, − 0.31]	[− 0.46, − 0.24]	[− 0.48, − 0.27]	[− 0.46, − 0.24]
	(0.000)	(0.000)	(0.000)	(0.000)	(0.000)	(0.000)	(0.000)	(0.000)	(0.000)	(0.000)	(0.000)	(0.000)
		− 1.12										
hs-cTnT at admission		[− 2.30, 0.06]										
		(0.026)										
			− 0.78									
hs-cTnT 8 h after PCI			[− 1.10, − 0.46]									
			(0.000)									
				− 0.88								
hs-cTnT 16 h after PCI				[− 1.28, − 0.48]								
				(0.000)								
					− 1.22							
hs-cTnT 24 h after PCI					[− 1.70, − 0.73]							
					(0.000)							
						0.00						
hs-cTnT 48 h after PCI						[− 0.00, 0.00]						
						(0.175)						
							− 1.19					
hs-cTnT 72 h after PCI							[− 2.01, − 0.37]					
							(0.005)					
								0.00				
Peak hs-cTnT								[− 0.00, 0.00]				
								(0.161)				
									− 0.16			
LGE mass									[− 0.27, − 0.06]			
									(0.002)			
										− 0.22		
GZF mass										[− 0.60, 0.17]		
										(0.272)		
											− 5.87	
MVO mass											[− 8.24, − 3.50]	
											(0.000)	
												− 3.37
Type of myocardial infarction												[− 5.59, − 1.15]
												(0.003)
Observations	176	176	172	166	163	147	94	176	176	176	160	176
*R*^2^	0.168	0.185	0.278	0.234	0.271	0.182	0.173	0.180	0.212	0.174	0.256	0.226
Adjusted *R*^2^	0.164	0.176	0.270	0.225	0.262	0.171	0.155	0.171	0.203	0.165	0.247	0.217

Coefficients; 95% confidence intervals in brackets; *p* values in parentheses

*CMR* cardiac magnetic resonance, *GZF* greyzone fibrosis, *L*V*EF* left ventricular ejection fraction, *hs-cTnT* high-sensitive cardiac Troponin T, *LGE* late gadolinium enhancement, *MI* myocardial infarction, *MVO* microvascular obstruction, *PCI* percutaneous coronary intervention

## Discussion

To the best of our knowledge, our study is the first to evaluate an association between GZF and the parameters MI-free survival and improvement of the LVEF in the long-term follow-up after acute myocardial infarction. In our analyses, hs-cTnT and LGE measurements revealed the best correlation with these endpoints. GZF failed as suitable predictor.

Ventricular arrhythmias and the development of ischemic heart disease are the most common complications of a MI and represent a relevant health burden due to hospitalization of the patients and costs for drug therapy or devices such as ICDs [9, 10]. Thus, individualized therapy is required, starting with the coronary intervention in acute MI and continuing through to follow-up care [10]. As research regarding MI is evolving, e.g., a new MI classification based on the different mechanism of tissues injury in MI [11], an individualized risk stratification after MI including biomarkers as well as imaging methods is needed. Currently, biomarkers such as hs-cTnT and LVEF are routinely used to assess the risk for post-MI events [2]. Previous studies demonstrated a strong correlation between troponin levels and a lower LVEF in the follow-up [12]. These results were reproducible in our study.

However, the accuracy of biomarkers and echocardiography are often limited by patient individual factors such as renal insufficiency (influencing troponin levels) or poor image quality due to adiposity.

CMR enables a reliable assessment of cardiac function, cardiac structure (e.g., scar development or microvascular obstruction), as well as the detection of left ventricular thrombi and has shown promising results for estimating the individual risk of complications after MI [10, 13]. Yet, CMR methods post-MI vary widely and CMR is not routinely recommended after MI [10]. Given the high costs and the low availability, a targeted use in patients who will benefit most is desirable. LVEF assessed by CMR, assessment of intramural hemorrhage and LGE quantification are known as most valuable analyses after MI with good correlations with all-cause mortality or heart failure-driven hospitalization and with biomarkers [14–18]. This is in line with our findings that LGE had a good correlation with MI-free survival and improvement in LVEF. In our analyses, the presence of STEMI (in contrast to NSTEMI) showed a correlation with the improvement in LVEF. This is most likely attributed to a greater infarct size and area at risk in STEMI patients, which has been described previously [19].

The assessment of LVEF by CMR has been reported to provide excellent reliability [20]. We were able to report a similar interobserver variability for our LVEF measurements indicating a high consistency across all patients.

In contrast, the assessment of myocardial edema and especially the GZF have been discussed controversially [15]. One reason for this might be the inconsistency in methodology based on different signal intensities attributed to GZF [6, 15].

Small studies have shown an association between GZF and ventricular arrhythmias in patients with previous MI and LVEF < 35% [21–24].

A large previous study of 979 patients with chronic coronary syndrome showed an association of the myocardial fibrosis and GZF with ventricular arrhythmias and sudden cardiac death—independent of LVEF [6]. Another study described GZF as none superior with regard to diagnostic accuracy over LGE [22].

In contrast to these data, GZF showed neither correlation with improvement of the LVEF nor with MI-free survival in our study. Hence, our data make GZF quantification seem dispensable after MI.

## Limitations

Several limitations of our study must be considered. First, we report on a retrospective study. The limited size of the study cohort and the small amount of second MI during follow-up restrict the power of the analyses. Thus, we cannot exclude an existing minor association. However, as other parameters showed a good prediction of our endpoints, we assume that potential minor associations can be neglected. Furthermore, we were not able to include death as an endpoint due to the low events. Since we report a retrospective study from 2014 to 2019, we cannot analyze T1 and T2 mapping data, as these images were not acquired at our institution during that period. Furthermore, we did not analyze intramural hemorrhage. Mean time between CMR 1 and CMR 2 varied to up to 1 year due to logistical reasons, representing routine clinical processes. Nevertheless, since our findings were consistent, we assume that there was no significant impact on our main results. The high number of re-hospitalizations can be attributed to these patients either undergoing staged coronary intervention for residual stenosis or undergoing follow-up coronary angiography after intervention for acute myocardial infarction, both of which were standard practices at our institution during this period.

## Conclusion

The greyzone fibrosis analysis does not add predictive value of CMR in patients after MI, as it shows worse correlation compared to hs-cTnT and LGE regarding the MI-free survival. Furthermore, GZF is not suitable as predictor of improvement of LVEF.

## Supplementary Information

Below is the link to the electronic supplementary material.

Supplementary file1 (DOCX 17 KB)

Supplementary file2 (DOCX 17 KB)

Supplementary file3 (DOCX 17 KB)

Supplementary file4 (DOCX 18 KB)

## Data Availability

The data are not publicly available due to containing information that could compromise the privacy of the research participants.
