# Supplementary material for: Correlation of greyzone fibrosis compared to troponin T and late gadolinium enhancement with survival and ejection fraction in patients after acute myocardial infarction
Source: Clin Res Cardiol. 2024 Sep 4;114(6):749–59. doi: 10.1007/s00392-024-02536-w (PMC12089158; doi:10.1007/s00392-024-02536-w)
Supplement: Supplementary file 1 — Supplementary file1 (DOCX 17 KB) [file 392_2024_2536_MOESM1_ESM.docx]

Results of multivariable cox models on the endpoint MI-free survival

|  | 1 | 2 | 3 | 4 | 5 | 6 | 7 | 8 | 9 | 10 |
| --- | --- | --- | --- | --- | --- | --- | --- | --- | --- | --- |
| hs-cTnT at admission | 1.87 |  |  |  |  |  |  |  |  |  |
|  | [1.18,2.97] |  |  |  |  |  |  |  |  |  |
|  | (0.008) |  |  |  |  |  |  |  |  |  |
| Greyzone mass | 1.09 | 1.16 | 1.16 | 1.12 | 1.17 | 1.19 | 1.16 | 1.16 | 1.18 | 1.20 |
|  | [0.94,1.27] | [0.98,1.36] | [0.99,1.35] | [0.95,1.33] | [0.98,1.39] | [1.00,1.42] | [0.99,1.36] | [0.98,1.36] | [0.98,1.41] | [0.98,1.46] |
|  | (0.252) | (0.077) | (0.073) | (0.180) | (0.074) | (0.052) | (0.066) | (0.080) | (0.075) | (0.077) |
| hs-cTnT 8 hours after PCI |  | 1.00 |  |  |  |  |  |  |  |  |
|  |  | [0.80,1.25] |  |  |  |  |  |  |  |  |
|  |  | (0.993) |  |  |  |  |  |  |  |  |
| hs-cTnT 16 hours after PCI |  |  | 1.07 |  |  |  |  |  |  |  |
|  |  |  | [0.82,1.39] |  |  |  |  |  |  |  |
|  |  |  | (0.633) |  |  |  |  |  |  |  |
| hs-cTnT 24 hours after PCI |  |  |  | 1.19 |  |  |  |  |  |  |
|  |  |  |  | [0.90,1.57] |  |  |  |  |  |  |
|  |  |  |  | (0.226) |  |  |  |  |  |  |
| hs-cTnT 48 hours after PCI |  |  |  |  | 1.00 |  |  |  |  |  |
|  |  |  |  |  | [1.00,1.00] |  |  |  |  |  |
|  |  |  |  |  | (0.955) |  |  |  |  |  |
| hs-cTnT 72 hours after PCI |  |  |  |  |  | 0.91 |  |  |  |  |
|  |  |  |  |  |  | [0.56,1.49] |  |  |  |  |
|  |  |  |  |  |  | (0.703) |  |  |  |  |
| peak hs-cTnT |  |  |  |  |  |  | 1.00 |  |  |  |
|  |  |  |  |  |  |  | [0.99,1.01] |  |  |  |
|  |  |  |  |  |  |  | (0.977) |  |  |  |
| LGE mass |  |  |  |  |  |  |  |  | 1.00 |  |
|  |  |  |  |  |  |  |  |  | [0.95,1.06] |  |
|  |  |  |  |  |  |  |  |  | (0.914) |  |
| MVO |  |  |  |  |  |  |  |  |  | 0.77 |
|  |  |  |  |  |  |  |  |  |  | [0.09,6.41] |
|  |  |  |  |  |  |  |  |  |  | (0.812) |
| Observations | 176 | 172 | 166 | 163 | 147 | 94 | 176 | 176 | 174 | 160 |

Hazard rations; 95% confidence intervals in brackets; p-values in parentheses.

hs-cTnT high sensitive Troponin T, LGE Late Gadolinium Enhancement, MVO microvascular obstruction, PCI percutaneous coronary intervention
