# Supplementary material for: Correlation of greyzone fibrosis compared to troponin T and late gadolinium enhancement with survival and ejection fraction in patients after acute myocardial infarction
Source: Clin Res Cardiol. 2024 Sep 4;114(6):749–59. doi: 10.1007/s00392-024-02536-w (PMC12089158; doi:10.1007/s00392-024-02536-w)
Supplement: Supplementary file 2 — Supplementary file2 (DOCX 17 KB) [file 392_2024_2536_MOESM2_ESM.docx]

Results of multivariable cox models on the endpoint MI-free survival

|  | 1 | 2 | 3 | 4 | 5 | 6 | 7 | 8 | 9 | 10 |
| --- | --- | --- | --- | --- | --- | --- | --- | --- | --- | --- |
| hs-cTnT at admission | 2.02 |  |  |  |  |  |  |  |  |  |
|  | [1.31,3.12] |  |  |  |  |  |  |  |  |  |
|  | (0.001) |  |  |  |  |  |  |  |  |  |
| sex | 2.54 | 1.75 | 1.74 | 2.42 | 2.77 | 2.91 | 1.81 | 1.78 | 1.70 | 2.01 |
|  | [0.46,14.07] | [0.34,9.06] | [0.33,9.03] | [0.43,13.56] | [0.46,16.60] | [0.47,17.82] | [0.35,9.33] | [0.35,9.17] | [0.32,9.01] | [0.37,10.99] |
|  | (0.287) | (0.506) | (0.512) | (0.314) | (0.264) | (0.248) | (0.478) | (0.491) | (0.532) | (0.419) |
| hs-cTnT 8 hours after PCI |  | 1.04 |  |  |  |  |  |  |  |  |
|  |  | [0.86,1.27] |  |  |  |  |  |  |  |  |
|  |  | (0.667) |  |  |  |  |  |  |  |  |
| hs-cTnT 16 hours after PCI |  |  | 1.12 |  |  |  |  |  |  |  |
|  |  |  | [0.88,1.42] |  |  |  |  |  |  |  |
|  |  |  | (0.376) |  |  |  |  |  |  |  |
| hs-cTnT 24 hours after PCI |  |  |  | 1.23 |  |  |  |  |  |  |
|  |  |  |  | [0.95,1.60] |  |  |  |  |  |  |
|  |  |  |  | (0.115) |  |  |  |  |  |  |
| hs-cTnT 48 hours after PCI |  |  |  |  | 1.00 |  |  |  |  |  |
|  |  |  |  |  | [1.00,1.00] |  |  |  |  |  |
|  |  |  |  |  | (0.934) |  |  |  |  |  |
| hs-cTnT 72 hours after PCI |  |  |  |  |  | 1.05 |  |  |  |  |
|  |  |  |  |  |  | [0.68,1.60] |  |  |  |  |
|  |  |  |  |  |  | (0.839) |  |  |  |  |
| peak hs-cTnT |  |  |  |  |  |  | 1.00 |  |  |  |
|  |  |  |  |  |  |  | [1.00,1.00] |  |  |  |
|  |  |  |  |  |  |  | (0.948) |  |  |  |
| LGE mass |  |  |  |  |  |  |  | 1.02 |  |  |
|  |  |  |  |  |  |  |  | [0.97,1.08] |  |  |
|  |  |  |  |  |  |  |  | (0.343) |  |  |
| Greyzone mass |  |  |  |  |  |  |  |  | 1.15 |  |
|  |  |  |  |  |  |  |  |  | [0.99,1.34] |  |
|  |  |  |  |  |  |  |  |  | (0.071) |  |
| MVO |  |  |  |  |  |  |  |  |  | 1.60 |
|  |  |  |  |  |  |  |  |  |  | [0.29,8.77] |
|  |  |  |  |  |  |  |  |  |  | (0.585) |
| Observations | 176 | 172 | 166 | 163 | 147 | 94 | 176 | 174 | 176 | 160 |

Hazard rations; 95% confidence intervals in brackets; p-values in parentheses.

hs-cTnT high sensitive Troponin T, LGE Late Gadolinium Enhancement, MVO microvascular obstruction, PCI percutaneous coronary intervention
