# Supplementary material for: Correlation of greyzone fibrosis compared to troponin T and late gadolinium enhancement with survival and ejection fraction in patients after acute myocardial infarction
Source: Clin Res Cardiol. 2024 Sep 4;114(6):749–59. doi: 10.1007/s00392-024-02536-w (PMC12089158; doi:10.1007/s00392-024-02536-w)
Supplement: Supplementary file 3 — Supplementary file3 (DOCX 17 KB) [file 392_2024_2536_MOESM3_ESM.docx]

Results of multivariable cox models on the endpoint MI-free survival

|  | 1 | 2 | 3 | 4 | 5 | 6 | 7 | 9 | 10 | 12 |
| --- | --- | --- | --- | --- | --- | --- | --- | --- | --- | --- |
| hs-cTnT at admission | 1.93 |  |  |  |  |  |  |  |  |  |
|  | [1.24,3.02] |  |  |  |  |  |  |  |  |  |
|  | (0.004) |  |  |  |  |  |  |  |  |  |
| type of myocardial infarction | 1.04 | 0.52 | 0.45 | 0.24 | 0.84 | 0.53 | 0.70 | 0.57 | 0.60 | 0.85 |
|  | [0.21,5.20] | [0.10,2.70] | [0.08,2.51] | [0.04,1.55] | [0.14,5.04] | [0.08,3.41] | [0.16,3.15] | [0.12,2.64] | [0.13,2.78] | [0.15,4.89] |
|  | (0.959) | (0.435) | (0.365) | (0.134) | (0.850) | (0.504) | (0.644) | (0.473) | (0.517) | (0.857) |
| hs-cTnT 8 hours after PCI |  | 1.07 |  |  |  |  |  |  |  |  |
|  |  | [0.87,1.32] |  |  |  |  |  |  |  |  |
|  |  | (0.507) |  |  |  |  |  |  |  |  |
| hs-cTnT 16 hours after PCI |  |  | 1.17 |  |  |  |  |  |  |  |
|  |  |  | [0.90,1.52] |  |  |  |  |  |  |  |
|  |  |  | (0.251) |  |  |  |  |  |  |  |
| hs-cTnT 24 hours after PCI |  |  |  | 1.33 |  |  |  |  |  |  |
|  |  |  |  | [1.01,1.75] |  |  |  |  |  |  |
|  |  |  |  | (0.039) |  |  |  |  |  |  |
| hs-cTnT 48 hours after PCI |  |  |  |  | 1.00 |  |  |  |  |  |
|  |  |  |  |  | [1.00,1.00] |  |  |  |  |  |
|  |  |  |  |  | (0.944) |  |  |  |  |  |
| hs-cTnT 72 hours after PCI |  |  |  |  |  | 1.05 |  |  |  |  |
|  |  |  |  |  |  | [0.68,1.62] |  |  |  |  |
|  |  |  |  |  |  | (0.831) |  |  |  |  |
| peak hs-cTnT |  |  |  |  |  |  | 1.00 |  |  |  |
|  |  |  |  |  |  |  | [1.00,1.00] |  |  |  |
|  |  |  |  |  |  |  | (0.949) |  |  |  |
| LGE mass |  |  |  |  |  |  |  | 1.03 |  |  |
|  |  |  |  |  |  |  |  | [0.98,1.08] |  |  |
|  |  |  |  |  |  |  |  | (0.292) |  |  |
| Greyzone mass |  |  |  |  |  |  |  |  | 1.11 |  |
|  |  |  |  |  |  |  |  |  | [1.00,1.23] |  |
|  |  |  |  |  |  |  |  |  | (0.060) |  |
| MVO |  |  |  |  |  |  |  |  |  | 1.65 |
|  |  |  |  |  |  |  |  |  |  | [0.29,9.49] |
|  |  |  |  |  |  |  |  |  |  | (0.572) |
| Observations | 176 | 172 | 166 | 163 | 147 | 94 | 176 | 174 | 176 | 160 |

Hazard rations; 95% confidence intervals in brackets; p-values in parentheses.

hs-cTnT high sensitive Troponin T, LGE Late Gadolinium Enhancement, MVO microvascular obstruction, PCI percutaneous coronary intervention
