# Supplementary material for: Correlation of greyzone fibrosis compared to troponin T and late gadolinium enhancement with survival and ejection fraction in patients after acute myocardial infarction
Source: Clin Res Cardiol. 2024 Sep 4;114(6):749–59. doi: 10.1007/s00392-024-02536-w (PMC12089158; doi:10.1007/s00392-024-02536-w)
Supplement: Supplementary file 4 — Supplementary file4 (DOCX 18 KB) [file 392_2024_2536_MOESM4_ESM.docx]

Results of multivariable linear regression models on the endpoint delta LVEDV (=LVEDV CMR 2 minus LVEDV CMR 1)

|  | 1 | 2 | 3 | 4 | 5 | 6 | 7 | 8 | 10 | 11 | 13 |
| --- | --- | --- | --- | --- | --- | --- | --- | --- | --- | --- | --- |
| delta LVEDV | -0.32 | -0.33 | -0.37 | -0.38 | -0.36 | -0.33 | -0.27 | -0.32 | -0.37 | -0.35 | -0.33 |
|  | [-0.43,-0.21] | [-0.44,-0.22] | [-0.48,-0.26] | [-0.49,-0.27] | [-0.47,-0.24] | [-0.46,-0.21] | [-0.42,-0.13] | [-0.43,-0.21] | [-0.48,-0.26] | [-0.46,-0.24] | [-0.45,-0.20] |
|  | (0.000) | (0.000) | (0.000) | (0.000) | (0.000) | (0.000) | (0.000) | (0.000) | (0.000) | (0.000) | (0.000) |
| type of myocardial infarction | 4.23 |  |  |  |  |  |  |  |  |  |  |
|  | [-5.98,14.44] |  |  |  |  |  |  |  |  |  |  |
|  | (0.414) |  |  |  |  |  |  |  |  |  |  |
| hs-cTnT at admission |  | 4.98 |  |  |  |  |  |  |  |  |  |
|  |  | [-0.29,10.25] |  |  |  |  |  |  |  |  |  |
|  |  | (0.064) |  |  |  |  |  |  |  |  |  |
| hs-cTnT 8 hours after PCI |  |  | 3.66 |  |  |  |  |  |  |  |  |
|  |  |  | [2.21,5.11] |  |  |  |  |  |  |  |  |
|  |  |  | (0.000) |  |  |  |  |  |  |  |  |
| hs-cTnT 16 hours after PCI |  |  |  | 4.60 |  |  |  |  |  |  |  |
|  |  |  |  | [2.63,6.57] |  |  |  |  |  |  |  |
|  |  |  |  | (0.000) |  |  |  |  |  |  |  |
| hs-cTnT 24 hours after PCI |  |  |  |  | 4.48 |  |  |  |  |  |  |
|  |  |  |  |  | [2.12,6.83] |  |  |  |  |  |  |
|  |  |  |  |  | (0.000) |  |  |  |  |  |  |
| hs-cTnT 48 hours after PCI |  |  |  |  |  | -0.00 |  |  |  |  |  |
|  |  |  |  |  |  | [-0.00,0.00] |  |  |  |  |  |
|  |  |  |  |  |  | (0.630) |  |  |  |  |  |
| hs-cTnT 72 hours after PCI |  |  |  |  |  |  | 7.19 |  |  |  |  |
|  |  |  |  |  |  |  | [3.73,10.64] |  |  |  |  |
|  |  |  |  |  |  |  | (0.000) |  |  |  |  |
| peak hs-cTnT |  |  |  |  |  |  |  | -0.00 |  |  |  |
|  |  |  |  |  |  |  |  | [-0.00,0.00] |  |  |  |
|  |  |  |  |  |  |  |  | (0.661) |  |  |  |
| LGE mass |  |  |  |  |  |  |  |  | 0.86 |  |  |
|  |  |  |  |  |  |  |  |  | [0.45,1.28] |  |  |
|  |  |  |  |  |  |  |  |  | (0.000) |  |  |
| Greyzone mass |  |  |  |  |  |  |  |  |  | 1.28 |  |
|  |  |  |  |  |  |  |  |  |  | [0.29,2.26] |  |
|  |  |  |  |  |  |  |  |  |  | (0.011) |  |
| MVO |  |  |  |  |  |  |  |  |  |  | 14.56 |
|  |  |  |  |  |  |  |  |  |  |  | [2.45,26.66] |
|  |  |  |  |  |  |  |  |  |  |  | (0.019) |
| Observations | 176 | 176 | 172 | 166 | 163 | 147 | 94 | 176 | 174 | 176 | 160 |
| *R*^2^ | 0.162 | 0.179 | 0.270 | 0.273 | 0.228 | 0.165 | 0.260 | 0.163 | 0.239 | 0.193 | 0.158 |
| Adjusted *R*^2^ | 0.153 | 0.165 | 0.257 | 0.260 | 0.213 | 0.147 | 0.235 | 0.149 | 0.226 | 0.179 | 0.142 |

Coefficients; 95% confidence intervals in brackets; p-values in parentheses.

hs-cTnT high sensitive Troponin T, LGE Late Gadolinium Enhancement, LVEDV left ventricular end-diastolic volume, MVO microvascular obstruction, PCI percutaneous coronary intervention
